# ‘Finally, in Hands I Can Trust’: Perspectives on Trust in Motor Neurone Disease Care

**DOI:** 10.3390/healthcare13161994

**Published:** 2025-08-14

**Authors:** Dominika Lisiecka, Neil Dyson, Keith Malpress, Anthea Smith, Ellen McNeice, Peter Shack, Karen Hutchinson

**Affiliations:** 1 Department of Nursing and Healthcare Sciences, Munster Technological University–Kerry Campus, V92 CX88 Tralee, Ireland; 2 Kerry SLT Clinic, Scotia Clinic, Manor West, V92 AT1Y Tralee, Ireland; 3 MND Australia, Lived Experience Network, Canberra, ACT 2606, Australia; 4 School of Population Health, University of New South Wales, Sydney, NSW 2052, Australia; 5 Central Coast Local Health District, Gosford, NSW 2250, Australia; 6 Australian Institute of Health Innovation, Macquarie University, Sydney, NSW 2109, Australia; 7 Central Coast Research Institute for Integrated Care, University of Newcastle, Gosford, NSW 2250, Australia

**Keywords:** motor neurone disease, amyotrophic lateral sclerosis, trust, integrated care, person-centred care

## Abstract

Integrated multidisciplinary care is recognised as essential for people living with motor neurone disease (PlwMND) and their families. The values underpinning integrated care, such as person-centredness, respect, empowerment, and co-production, are central to delivering meaningful and comprehensive support. Trust is an essential yet often overlooked element of effective person- and family-centred integrated care, particularly for PlwMND. While specialist multidisciplinary MND clinics represent the benchmark for evidence-based care, many PlwMND and their families depend significantly on local and community-based support services to maintain quality of life. Trust directly influences their engagement with these services and the continuity of care provided. Trust enables understanding of personal priorities and how they change as the disease progresses, ultimately allowing for person-centred care to happen. Trust is necessary to enable service co-production, which is a strong value of integrated care. Research highlights seven key domains of support essential to PlwMND and their carers: practical, social, informational, psychological, physical, emotional, and spiritual. Effective integrated care requires strong relationships built upon trust, shared decision-making, respect for individuality, and clear communication. Furthermore, due to the rapidly progressive nature of MND, care priorities and perceived symptom burdens may shift significantly over short periods, making flexible, temporally sensitive approaches critical. A dynamic, inclusive model of decision-making that fosters autonomy within and regular co-review of needs is recommended. This perspective paper examines how person- and family-centred integrated care is currently being delivered, what is working well, and how these practices can be further strengthened to enhance the care experiences of PlwMND, their families, and the health and social care providers involved. This paper builds on both theoretical knowledge and clinical experience to offer our perspective on the critical role of trust in co-producing integrated care for PlwMND. It brings together the voices of clinicians and researchers, alongside those with lived experience of MND. We propose a diagram of care that embeds the core values of integrated, person-centred care within the specific context of MND. Our aim is to enhance collaborative practices, strengthen cross-sector partnerships, and ultimately improve the care experiences for professionals, PlwMND, and their families.

## 1. Introduction

Motor neurone disease (MND) is a rare, multisystem, incurable, and progressive illness. Integrated multidisciplinary care is recognised as essential for people living with MND (PlwMND) and their families [1,2]. The values underpinning integrated care, such as person-centredness, respect, empowerment, and co-production, are central to delivering meaningful and comprehensive support [3].

Person-centred care is a well-known concept, but its definition and practical application remain unclear [4,5]. Key aspects of person-centred care include holistic care, respect, value, dignity, choice, self-determination, and purposeful living, which shifts focus from a purely biomedical model to one that emphasises individual autonomy and the right to make own decisions [6]. Despite its appeal, implementing person-centred care is challenging due to factors such as staff attitudes, limited time, patient vulnerability, and professionals’ belief that they already practice it [4]. Although person-centred care provides numerous benefits for both patients and healthcare professionals, such as enhancing patient involvement, promoting empowerment, and supporting self-advocacy, gaps in our understanding of the implementation of person-centred care are still present [5].

At its core, trust is the belief that individuals and institutions will act appropriately, perform competently and responsibly, and consider the interests of the trustors in their actions [7,8]. Trust is an essential yet often overlooked element that influences how PlwMND engage in healthcare [9] and the effectiveness of person- and family-centred integrated care [10,11,12]. While specialist multidisciplinary MND clinics represent the benchmark for evidence-based care [13,14], many PlwMND and their families depend significantly on local and community-based support services to address care needs and maintain quality of life.

The knowledge of non-MND specialist Health and Social Care Professionals (HSCPs) remains a persistent concern for PlwMND and their families, influencing their perception of HSCPs’ ability to provide appropriate services and support [1]. Beyond competency, the quality of interactions between PlwMND, their families, and HSCPs shapes their expectations of the relationship [9]. When HSCPs demonstrate empathy, kindness, and authenticity, they foster trust, whereas a lack of understanding can weaken trust or lead to distrust in the ability of or information provided by HSCPs [8,9,15,16]. These observations indicate that trust not only boosts engagement with services but also ensures PlwMND and their families feel supported in retaining control over their lives and care [16]. Trust plays a crucial role in comprehending personal and family values and priorities as they shift with disease progression, thereby enabling person-centred care [16,17].

An often overlooked but vital aspect of building trusting relationships, as highlighted in a recent review, is the early identification and effective management of cognitive and behavioural changes, which can impact up to 50% of PlwMND [18,19]. These changes can significantly shape the delivery of person-centred care and the lived experiences of families. Recognising cognitive changes early provide PlwMND and families invaluable time to connect meaningfully with HSCPs, fostering stronger relationships and enabling them to participate more actively in planning their care that aligns with their needs and hopes [18,20]. Cultivating relationships with HSCPs who understand their circumstances and values, and who implement strategies that support PlwMND to engage in care planning and decision-making, helps alleviate anxieties and balance autonomy with truly person-centred care [21].

Ultimately, trust is essential for building and maintaining relationships and underpins service engagement and co-creation for better health outcomes and satisfaction with care, a cornerstone of integrated care [2,22]. Nonetheless, trust is inherently fragile and can change significantly based on individual and family experiences [15]. Trust plays a critical and defining role and shapes the care journey for PlwMND. This perspective paper, written by PlwMND, family carers, and clinical academics together, combines theoretical, clinical, and personal perspectives. The aim is to discuss the role of trust in enhancing collaborative practices between PlwMND, family carers, and HSCPs and ultimately improve care experiences and foster greater satisfaction for everyone involved.

## 2. Understanding Core Constructs of Trust

There are five core constructs of trust: three constructs that drive trust, authenticity, empathy, and competency [23,24], and two underpinning foundational constructs for building trust, vulnerability and reciprocity [8,25], identified in the literature as providing the fundamental conditions for building the foundations of trust. The three trust drivers together are mutually reinforcing, yet their significance may shift depending on care context, cultural, and personal experiences. Whilst competency presumes that HSCPs will uphold individuals’ best interests and safeguard confidentiality, it is the empathic, caring, and authentic actions of HSCPs that cultivate transparency, emotional presence, and non-judgement, ultimately making trust more felt and visible [23,26,27]. The relationships between the three trust drivers in real-world settings have been demonstrated in models such as the Trust Triangle [28,29] and the Trust Trifecta [30].

The two foundational trust constructs of vulnerability and reciprocity are consistently identified in the literature as essential precursors to trust, fostering mutual cooperation, collective benefit, and relational openness [8,15,23,25,30,31]. Together, these factors foster greater satisfaction, safety, and connectedness in the healthcare relationship. The core values related to trust building identified are Compassion and Kindness, Dignity and Respect, Holistic, Partnership, and Coproduction [32]. These values are well aligned with the principles of integrated care [33]. Table 1 presents a list of terms and short definitions to provide more context to our topic.

There are many models of trust, but as highlighted by Taylor et al. (2023), there still remains limited knowledge on how to build and measure trust [8]. Mayer’s et al. (1995) Integrative Model of Organisational Trust is helpful to understand how trust is developed and maintained in relationships, especially in organisational and interpersonal contexts [7]. In this model, trust is understood as the willingness of one party to be vulnerable to another, based on the expectation that the other will act beneficially or at least not harmfully. The willingness to take a risk is a behavioural expression of trust, which happens if there is perceived trustworthiness (linked to ability, benevolence, and integrity).

A concept of “a House of Trust” illustrates how trust is built (co-created) over time and using multiple components. Apart from the central values presented above, there is a need for multiple support structures to develop person-centred and family-centred services [32]. The four support pillars in this model are Transparency, Communication, Dignity and Respect, and Resilience.

## 3. Trust and MND—From Our Personal Perspectives

The authors discussed their own experiences and perspectives of trust in the context of MND to identify the main issues arising and the importance of receiving/delivering truly integrated and person-centred care for everyone living with this condition.

## 4. Engagement and Co-Production

Information about this perspective paper was disseminated via the MND Australia Lived Experience Network and Kerry Specialist Palliative Care Unit in Ireland to generate interest in volunteering to share personal experiences and perspectives on trust in healthcare. Interested individuals contacted the clinical academics (D.L. and K.H.) directly by email or phone. The clinical academics were transparent about the paper’s context, goals, scope, and process, including co-author roles and potential outcomes as “co-creators of knowledge” and impact [44]. Contribution was voluntary, and interested individuals were given the choice to be named as authors or to remain anonymous. Snowball sampling [45] extended invitations through MND lived experience networks. A topic guide was distributed via email to generate discussions. Lived experience experts responded either in writing or verbally, depending on their preference and ability. There was no requirement to comment on all topics, but to describe personal perspectives important to them. The amount of information shared was entirely up to each individual. All perspectives were included and organised into broad thematic sections by the clinical academics. The drafts of thematic sections (Experiences Eroding Trust, Experiences Facilitating Trust, Trust as Foundation of Person-centred Integrated Care in MND, and Conclusions) were disseminated by email to all the authors for their feedback and comments. K.H. and D.L. took the lead in drafting the paper until the final version was approved by everyone. A series of in-person and online meetings and email exchanges took place over May–June 2025 as this paper was being drafted. Each co-author provided input and feedback to ensure that their perspectives were conveyed clearly and accurately whilst maintaining anonymity. The main points from our discussions and analysis are summarised below.

## 5. Experiences Eroding Trust

For many individuals, the beginning of the MND journey is a highly traumatic period, often marked by a prolonged and challenging diagnostic process involving multiple service providers, many with limited knowledge of MND, recurrent misdiagnosis, and significant uncertainty. For many PlwMND, showing vulnerability early can feel risky, especially when trust in HSCPs has not been established. This can add to their emotional, organisational, and financial challenges when engaging with HSCPs and navigating a fragmented healthcare system. Even when trust has been established with HSCPs previously, such as a General Practitioner, their limited understanding and knowledge of MND, particularly in their “frontline” role, can strain relationships during the diagnosis phase. As a result, trust in HSCPs may be challenged from the outset of the MND journey. Yet, for many PlwMND, establishing a trusting relationship with a HSCP or service who understand MND is often perceived as a significant turning point, marking a transition toward greater stability, safety, and support. For some PlwMND, identifying even a single trust companion (a HSCP whom they feel they can trust) can bring significant value and reassurance. This relationship can serve as an emotional anchor while navigating multiple services and interacting with numerous HSCPs, often in complex and overwhelming circumstances. The concept of trust companionship goes beyond routine care, as it reflects a deeper, human connection in which the HSCP has the potential to greatly enhance the experience of care.

As MND progresses without offering any remission periods, time becomes very precious. Delays, service fragmentation, and administrative errors (such as incorrect appointment times or dates) can result in the loss of valuable time for PlwMND, for whom timely access and delivery of care is critical. Access to specialised MND clinics varies, creating disparities in care experiences. While multidisciplinary input is essential for effective symptom management, future care and support planning, and timely provision of equipment, not all individuals have access to knowledgeable and experienced HSCPs. Consequently, the quality of care often hinges on the availability and accessibility of local resources, influencing the development of trust. For PlwMND, service stability and predictability are also vital, placing high value on seeing the same HSCPs over time. Any service changes, including being discharged from particular disciplines as the disease progresses, must be carefully planned and effectively communicated. Consistency is vital—understanding me as a person, my disease and its progression, and creating and maintaining a record/history.

A lack of reciprocal, two-way communication between PlwMND and HSCPs can significantly undermine the development of trust within the therapeutic relationship. Some HSCPs may believe they are listening, yet their responses and non-verbal cues sometimes reveal a lack of genuine attentiveness or presence, suggesting they are not fully hearing or engaging in what is being said. One observed example involves a HSCP who, while engaging with a PlwMND, typed notes during the conversation. Although the need to collect clinical information is understandable, this practice was perceived as distracting and potentially disengaging. In some cases, it appeared that HSCPs were more focused on preparing their own responses rather than actively listening to the individual’s concerns. Another example is withholding the outcome of medical assessment or test results, which can create the impression that important information is being deliberately concealed and creates room for apprehension. In contrast, when professionals demonstrated active listening skills, adopted a transparent approach, and took time for explanation and reflection, these attentive and mutually responsive interactions were observed to significantly enhance trust. Such encounters highlight that active and empathetic listening is a key factor in fostering emotional safety and in supporting the development of meaningful, open therapeutic relationships. As MND progresses, clear, honest communication is crucial to making choices about the management options and service engagement.

Trust may be compromised when HSCPs are perceived as being focused primarily on the disease itself rather than looking at the person holistically, particularly when communication centres on clinical aspects and outcomes in isolation from the person’s lived experience. Generic reassurances such as “you are doing great” can be perceived as dismissive or invalidating, especially when the PlwMND is managing multiple, often distressing, symptoms.

The limited pool of experienced and consistently available HSCPs directly impacts the building and sustaining of trusting relationships with PlwMND. Addressing this challenge requires a stronger focus on workforce development, including improved training opportunities. These measures are essential for enhancing workforce competence, consistency of resources, and for strengthening the overall trustworthiness of services for PlwMND.

## 6. Experiences Facilitating Trust

Trust is a central and aspirational goal in the relationship between PlwMND and their HSCPs. While trust is deeply valued, it is rarely instantaneous and typically requires time and consistent and reciprocal efforts. Key contributors to the development of trust include the presence of empathy, clinical competence, and authenticity in interactions with HSCPs. However, these elements are not uniformly weighted, and their relative importance may vary from person to person. For some PlwMND, empathy and authenticity may be prioritised over clinical expertise during the development of trust. For others, demonstrable competence and experience in MND is paramount.

A key factor in establishing trust across all HSCPs is their ability to engage with individuals in an accepting, respectful, and non-condescending manner, while also demonstrating knowledge of MND. When such expertise is lacking, openly acknowledging this and making a genuine effort to develop their understanding is valued. Openness, “no ego”, and a commitment to fostering an equal partnership are essential components of maintaining trust over time.

Many PlwMND express a preference for a care approach that involves gentle guidance, rather than directive decision-making. They value the ability to make their own choices, including the right to defer decisions or reconsider previously made ones. This includes respecting choices even when they differ or conflict with the clinical evidence or others’ preferences. PlwMND are human beings, with feelings, understanding, and the ability to distinguish and interpret events happening to and around them, even if sometimes they do not voice it.

Trust in HSCPs is often not absolute, particularly when it comes to treatment recommendations. Some individuals describe a need to educate themselves alongside professional advice, emphasising that they are unlikely to accept or adhere to prescribed interventions without a clear personal understanding of their purpose, risks, and benefits. This highlights the importance of transparent communication and shared decision-making in fostering trust and supporting autonomy, reducing stress, reinforcing a sense of agency, dignity, and personhood throughout the MND journey.

Access to peer support is perceived as beneficial by some PlwMND and their partners, as it provides reassurance, shared understanding, and can enhance trust in the care process. For these individuals, connecting with others who have similar experiences fosters a sense of solidarity and emotional validation. However, this is not universal; others may find peer interactions distressing, particularly when exposed to individuals at more advanced stages of the disease, which may affect their willingness to engage with MND clinics. For some, such encounters may evoke fear or a sense of hopelessness, and if not acknowledged or appropriately supported, may in some cases diminish trust in HSCPs and disengage from services.

## 7. Trust as Foundation of Person-Centred Integrated Care in MND

**Figure 1:**
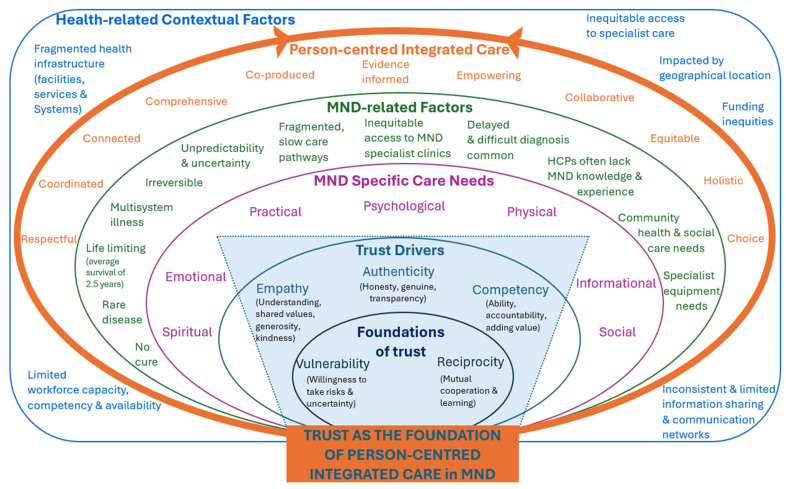
Trust as a foundation of person-centred integrated care for PlwMND (authors’ own figure). Abbreviations: MND—motor neurone disease; HSCPs—health and social care professionals.

It is our opinion, that trust forms a foundation of person-centred integrated care for PlwMND (Figure 1). The importance of the foundations of trust (vulnerability and reciprocity) and the trust drivers (empathy, authenticity, and competency) strongly emerged during our discussions. All foundational elements and trust drivers are essential in addressing the specific care needs of PlwMND across seven domains—physical, practical, psychological, emotional, informational, spiritual, and social [46] (Figure 1 in purple). Thus, ensuring a holistic approach that considers the person as a whole, well beyond the physical and cognitive symptoms of the disease. Prioritising trust-building behaviours throughout the clinical trajectory of MND is pivotal to supporting PlwMND, including those experiencing cognitive changes. This approach carefully considers changes in physical and cognitive capacity and ensures care remains relational, respectful, and consistently person-centred throughout [20].

While there are many other neuro-degenerative diseases, the trajectory of MND is complex and quite distinctive (Figure 1 in green), which poses unique challenges for HSCPs, organisations, and systems striving to deliver person-centred integrated service. These challenges, however, can be addressed when the principles [33] and values [3] of integrated care are applied consistently and across the continuum of care. From our perspective, eleven principles emerged as particularly strong facilitators of trust within the context of MND (Figure 1 in orange). Services must be coordinated, connected, comprehensive, collaborative, and co-produced with PlwMND in a way that supports their choice and considers personal context and logistic issues. Services must be evidence informed, holistic, and equitable. Everyone involved in the service co-production and delivery should feel respected and empowered. These principles and values should not only be embedded at the micro level of individual care interactions but also require broader organisational and policy support at the meso and macro levels to enable meaningful and sustained implementation [2].

Health-related contextual factors (Figure 1 in blue) such as fragmentation of services, delayed access to care, limited workforce capacity, competency and availability, or inconsistent and limited information sharing and communication networks, are symptomatic of institutional and systemic trust failures, and significant inhibitors of person-centred integrated care requiring system-level improvements to address the insufficiencies and fragmentation [2,23,47,48]. We advocate for equitable and coordinated service provision for every PlwMND, irrespective of their demographic background or socio-economic status. The co-production of truly person-centred services requires a sustained, collaborative effort grounded in mutual interpersonal trust between PlwMND and HSCPs [2].

## 8. Conclusions and Future Directions

Trust is foundational in the care of PlwMND. There is no person-centred care without trust, and there can be no meaningful integrated care without a person-centred approach. Building this trust relies on key enablers (the “drivers of trust”), including empathy, authenticity, and clinical competence. Importantly, it is not necessary for PlwMND to trust every HSCP equally; rather, trust built with a few key individuals can anchor the person’s confidence in the wider service.

From HSCPs’ perspectives, as we deepen our understanding of how best to support PlwMND, we also uncover the immense complexity of this condition and the profoundly individual nature of each person’s experience. Our responsibility as HSCPs is to cultivate trust in the most authentic way possible. Do not hesitate to work with PlwMND—if you bring authenticity and empathy, you are already starting this relationship from the right place, which is hugely valued by PlwMND and their families. Clinical competence will follow, through supervision, training, and reflective practice, which many recognise and support.

Working with people at such a vulnerable and challenging time in their lives is not just a task, it is a privilege. Yet person-centred care in MND is uniquely challenged by the progressive nature of the disease and the urgency imposed by time. A holistic focus on the person and not just the condition offers a guiding framework for care and trust building. Knowing the person must come first; clinical knowledge of MND alone is not sufficient. Competence and empathy are not opposing qualities, and they must coexist to deliver meaningful, compassionate care.

Trust is essential not only for continuity of care but also for enabling truly integrated, co-produced services. It takes time to build trust and continuous effort to sustain it. A system-wide approach to trust development is essential as it creates the broader context in which HSCPs and PlwMND interact. This environment can either support or undermine the formation of meaningful, trustful relationships. The presence or absence of systemic trust profoundly influences the experiences of both those delivering and receiving care, creating either a supportive or unfavourable care environment. We must do better in prioritising it. Trust is both a responsibility and a privilege—it can be overwhelming at times and may place the HSCPs in a position of vulnerability. Yet when trust is genuinely earned, it is a profound measure of success.

Importantly, MND services should never be withdrawn without a clear, planned transition. Abrupt discontinuation can irreparably damage the trust that has been carefully built. Identifying a key person (a trust companion) who remains consistently engaged with the PlwMND and their family throughout the care journey is an important consideration. Maintaining that relationship can hold the entire care experience together. Regardless of their health professional role, this individual could play a vital part in helping navigate service-related transitions and fostering continuity, stability, and relational trust.

PlwMND encounter multiple HSCPs in the course of the diagnosis process and beyond. Each of those interactions (or touchpoints) has the potential to either sustain and boost the inherent trust, or, conversely, to erode it over time. The overall level of trust a person develops is shaped by the cumulative impact of these encounters, and this balance will naturally vary from individual to individual, just as the experience of MND is different for each person. It is important to recognise that HSCPs are humans too, each with their own personal and professional contexts, which inevitably influence their ability to engage during these critical touchpoints. As such, the outcomes of these interactions—whether they reinforce or diminish trust—are shaped not only by systems and protocols, but also by the emotional and relational dynamics between individuals on both sides of care.

Building mutually trusting relationships should be acknowledged and celebrated as a meaningful marker of healthcare success. Trust is reciprocal and fosters emotional safety, sustains engagement, and empowers confidence in making choices and retaining a sense of control. We need to remain committed to continuous learning and adapting to the evolving and individual needs of PlwMND and strive for integrity, honesty, consistency, and compassion. It is essential to create an environment that facilitates the engagement and co-production of care where PlwMND are recognised for who they are and empowered to use their time as efficiently and meaningfully as possible. Trust needs to be recognised as a vital foundation of and mechanism for delivering truly person-centred integrated care. And when we get this right for PlwMND, we are likely to get it right for many others.

This perspective paper highlighted both the importance and the complexity of trust in MND. Trust is not static; it is shaped over time through a series of interactions, relationships, and systems of care. For PlwMND, whose journey involves profound vulnerability, uncertainty, and change, trust becomes an essential foundation of effective, person-centred care. Trust in healthcare is an area which needs further exploration. Advancing understanding in this space suggests a review of existing theoretical models, with the aim of developing an integrated framework that supports purposeful and sustainable trust building across healthcare systems and relationships. Further research is required to better understand how trust is developed, sustained, or lost over time, and how healthcare systems, teams, and individual clinicians can support more consistent and compassionate MND care, with ongoing consideration of the impacts of physical, cognitive, and behavioural deficits. This includes exploring how organisational structures, communication practices, and workforce dynamics influence the experience of trust from the perspective of PlwMND, their families, and HSCPs.

## Figures and Tables

**Table 1:** Important terms and definitions.

Important Terms	Definitions (Adapted by Authors)
Person-centred care	Focuses on understanding and responding to everyone’s unique needs, values, circumstances, and preferences. It emphasises shared decision-making, respect for individuality, holistic care, and open communication—ensuring that care is tailored to what matters most to the person receiving it [5,34].
Integrated care	Refers to coordinated strategies that aim to improve health outcomes by addressing fragmentation and fostering continuity across the care continuum—built on relationships of trust among providers, systems, and the people they serve [35,36].
Trust	A relational belief in the competence, integrity, and goodwill of another [7,8].
Authenticity	The quality of being genuine and aligned with one’s core values, demonstrated through consistency, sincerity, and respectful boundaries [28,37].
Competency	The capacity to apply knowledge, skills, and sound judgement to effectively fulfil a role or task [26,28].
Empathy	The capacity to emotionally understand and connect with another person’s experience—by sensing what they feel, seeing from their perspective, and responding with genuine care [28,38].
Kindness	Is an action that supports or uplifts another, as experienced and valued by the person receiving it [39,40]. Often described as doing good without expectations.
Compassion	Is the emotional capacity to recognise another’s suffering through relational understanding, accompanied by a genuine motivation to alleviate that suffering through meaningful action [41].
Vulnerability	Is the willingness to be emotionally open or uncertain, grounded in the belief that the other person will respond with care, respect, and dignity [42,43].
Reciprocity	Reciprocity is the mutual exchange of support, effort, or understanding that fosters cooperation and shared benefit between individuals or groups [25].

## Data Availability

Data sharing is not applicable to this perspective article.
